# Sick-leave decisions for patients with severe subjective health complaints presenting in primary care: A cross-sectional study in Norway, Sweden, and Denmark

**DOI:** 10.3109/02813432.2013.844412

**Published:** 2013-12

**Authors:** Silje Maeland, Erik L. Werner, Marianne Rosendal, Ingibjorg H. Jonsdottir, Liv H. Magnussen, Stein Atle Lie, Holger Ursin, Hege R. Eriksen

**Affiliations:** ^1^Uni Health, Uni Research, Bergen, Norway; ^2^Faculty of Health and Social Sciences, Bergen University College, Norway; ^3^Research Unit for General Practice, Uni Health, Uni Research, Bergen, Norway; ^4^Research Unit for General Practice, Department of Public Health, Aarhus University, Denmark; ^5^The Institute of Stress Medicine, Gothenburg, Sweden; ^6^Department of Global Public Health and Primary Care, University of Bergen, Norway; ^7^Department of Education and Public Health, University of Bergen, Norway

**Keywords:** Diagnosis, family practice, general practice, medically unexplained symptoms, Norway, primary health care, sick leave, somatoform disorder

## Abstract

**Objectives:**

The primary objective of this study was to explore whether general practitioners (GPs) in Norway, Sweden, and Denmark make similar or different decisions regarding sick leave for patients with severe subjective health complaints (SHC). The secondary objective was to investigate if patient diagnoses, the reasons attributed for patient complaints, and GP demographics could explain variations in sick leave decisions.

**Design:**

A cross-sectional study.

**Method:**

Video vignettes of GP consultations with nine different patients.

**Subjects:**

126 GPs in Norway, Sweden, and Denmark.

**Setting:**

Primary care in Norway, Sweden, and Denmark.

**Main outcome measure:**

Sick leave decisions made by GPs.

**Results:**

“Psychological” diagnoses in Sweden were related to lower odds ratio (OR) of granting sick leave than in Norway (OR = 0.07; 95% CI = 0.01–0.83) Assessments of patient health, the risk of deterioration, and their ability to work predicted sick leave decisions. Specialists in general medicine grant significantly fewer sick leaves than non-specialists.

**Conclusion:**

Sick-leave decisions made by GPs in the three countries were relatively similar. However, Swedish GPs were more reluctant to grant sick leave for patients with “psychological” diagnoses. Assessments regarding health-related factors were more important than diagnoses in sick-leave decisions. Specialist training may be of importance for sick-leave decisions.

Norway has higher rates of sick leave and disability pension compared with Sweden and Denmark. Decisions made by general practitioners (GPs) in Norway, Sweden, and Denmark regarding sick leave were explored using video vignettes showing patients with severe subjective health complaints (SHC).

- Overall, the sick leave decisions made by GPs in the three countries were relatively similar.
- Patients given a “psychological” diagnosis were less likely to receive sick leave in Sweden compared with Norway.
- GP decisions to issue sick leave were associated with interpretations of patients’ medical conditions, their health, and their ability to work.

## Introduction

Subjective health complaints (SHC) such as musculoskeletal pain, sleep problems, and feelings of depression are common [1], and account for a substantial proportion of encounters in general practice [2]. Some patients develop chronic and persistent complaints, and different terms have been used to describe those conditions that do not have an obvious pathology and are of unclear aetiology [3]. In this paper, we refer to these as “severe subjective health complaints”.

Patients with severe SHC account for approximately one in five consultations in primary health care [4,5] and for more than half of the long-term sick leave issued [6,7]. In most countries, issuing sickness certificates is a core task in general practice [8,9] but there are large differences among GPs in how to do this [10].

Sick-leave-related legislation and guidelines indicate that an appropriate diagnosis is a prerequisite for assessment of functional ability [11] and for sick leave recommendations [12]. In 2007, Sweden implemented guidelines, suggesting the length of sick leave based on diagnosis [12]. The diagnosis given by a GP in Denmark plays a decisive role when municipal case managers decide who is entitled to sick-leave benefits [13]. However, this practice has been questioned [14].

It has been postulated that costs related to sickness absence are higher in Norway than in Sweden and Denmark [15,16]. However, international comparisons are limited, and there are substantial variations in the methods and data presented [17].

The primary objective of this study was to explore whether GPs in Norway, Sweden, and Denmark make similar or different decisions regarding sick leave for patients with severe SHC. The secondary objective was to investigate if the diagnosis, the reasons the GPs attributed for the patients’ complaints, and GP demographics could explain variations in the GPs’ sick-leave decisions.

## Material and methods

A total of 126 GPs in Norway (n = 56), Sweden (n = 29), and Denmark (n = 41) participated in the study, watching nine video vignettes. All participants from Sweden and Denmark, and 34 of the participants from Norway were specialists in general medicine. There were relatively fewer women among the Norwegian GPs. The Danish GPs had fewer years of experience as GPs than their Norwegian and Swedish colleagues (Table I).

**Table I.** Demographic profile of the participating GPs in Norway, Sweden, and Denmark (n = 126) and between-countries differences (ANOVA).

	Norway n (%)	Sweden n (%)	Denmark n (%)	p-value
Female	20 (36)	16 (55)	27 (66)	0.01
Age:				0.09
≤ 40	15 (27)	5 (17)	5 (12)	
41–50	21 (37)	6 (21)	19 (46)	
≥ 51	20 (36)	17 (59)	17 (41)	
GP experience (years)				0.01
≤ 10	21 (37)	10 (34)	22 (54)	
11–15	12 (21)	4 (14)	9 (22)	
≥ 16	23 (41)	14 (48)	10 (24)	
Specialist in general medicine	36 (64)	26 (90)	40 (98)	< 0.01
No, or other medical speciality	5 (9)	11* (38)	–	< 0.01

Notes: P-values ≤ 0.05 were considered statistically significant. *These GPs were also specialists in general medicine. Norway n = 56, Sweden n = 29, Denmark n = 41.

In Norway, we recruited GPs through yhe Norwegian Medical Association, to participate in a 15-hour Continuing Medical Education (CME) course, free of charge. The Norwegian Medical Association approved the course, giving 15 points accredited to the GPs’ CME score (necessary for obtaining or maintaining status as a specialist in general medicine). In Sweden, the GPs were recruited through the Swedish Medical Association and the Institute of Stress Medicine website. They were reimbursed €500 to watch the vignettes and answer questionnaires, mostly outside working hours. In Denmark, CME groups in the region of Southern Denmark and Central Denmark region were invited to participate and each GP was reimbursed €360 for watching the vignettes and answering the questionnaire outside working hours prior to the CME group meetings where the vignettes were used as basis for discussion.

The vignettes were based on videotaping of 19 actual consultations between a GP and patients with severe SHC. The patients who were videotaped gave consent for use of the material for research and teaching purposes. The research team and a reference group of four GPs selected a purposive sample of nine consultations with variation in respect of the patients’ age, sex, and type of complaints. The consultations were transcribed verbatim, making movie scripts that could be used for dramatization. Information that could identify the patient was excluded or rewritten. One of the GPs in the research team played the role as the GP, and professional actors were recruited for the patient roles. The vignettes included an introduction from the GP in which the patients’ medical history was presented, plus information about previous patient investigations, and the clinical results of these investigations (Table II). The vignettes were in Norwegian and subtitled in Swedish or Danish – three very similar languages.

**Table II.** Description of the patients presented in the video vignettes, gender, age, demography, complaints, and self-assessment of disability.

Vignette	Gender, age	Demography	First complaint mentioned in consultation/principal complaint	Secondary complaints	Self-assessment of disability
1	♀ 25	Single, no children Interrupted secondary education Currently in rehabilitation program Several short-time jobs and sick-leave spells	General pain in the neck, the back and in arms Intense pain 24 hours per day, 7 days a week	Respiratory complaints, no objective findings of asthma or other known somatic disease Anxiety and depression periodically treated with antidepressants	Expresses hope to achieve ability to work, but needs substantial improvement in health conditions first
2	♂ 40	Married, two children Working offshore on oil platform as a mechanic – two weeks on, four weeks off work Several shorter periods of sick leave and two long spells (one year each)	Back and neck pain	Sleep disturbances due to pain Irritable bowel syndrome, skin eczema	The work is physically hard and provokes pain He does not see himself in this job until retirement, but the salary and long periods off work make him keep the job
3	♀ 53	Housewife for 20 years with five foster-care children in addition to two biological children The fostering years have ended and her income consequently also Bringing up foster children has been challenging due to narcotics and psychiatric disorders in the foster children	Generalized, widespread non-specific pain	Anxiety, non-insulin dependent diabetes, general fatigue, no energy left	She has not had any working experiences outside home for nearly 30 years She feels exhausted and wants to be left alone with no demands of working activity or qualification for work
4	♂ 37	Married, unknown number of children. Previously working offshore, but started as self-employed in construction	General intense fatigue	No other complaints but has read about CFS which he finds fits his problems Economic burdens due to poor benefit coverage as self-employed	No work capacity
5	♂ 42	Married, three children Works as formwork carpenter No previous history of sick leave A 12-year-old daughter with serious behavioural problems, she refuses to go to school, meets her parents with substantial aggression, runs away from home. The girl is enrolled in a behavioural training program with great demands of parents’ involvement	He feels physically and psychologically exhausted, afraid that he might collapse No energy left to deal with his daughter after work	No other complaints.	He needs time off to deal with his family problems The program is set for 3–4 months
6	♀ 37	No information on marital status or children Working in a kindergarten Previous four-month sick leave for same complaints was followed by no symptoms for one and a half years	Periodic numbness, staring like a toothache, followed by headache and a sensation of anesthesia on the right side of the body; things slips out of her hand Extensive medical examination has not proved any cause for the symptoms	No other complaints	Difficult to work with these complaints, unsure about sick leave
7	♀ 35	No information on marital status or children Working as teacher in primary school No previous sick leave history, no previous psychiatric or somatic disorder	Feeling tired, weak, doesn't get things done, struggling, powerless, sleep disturbances Relates the symptoms to work overload	No other complaints	She feels she may need time out from work
8	♂ 36	Married, two small children Working as teacher at comprehensive level Very active sports trainer, coaches a 1st division handball team No previous sick leave history, no previous psychiatric or somatic disorder Worried about possible serious illness despite negative examinations so far An affair a year ago bothers him a lot	Pain started in the jaw muscle, following in the neck, head, and stomach	Sleep disturbances, frustrated, lack of energy, withdrawal from social events and friends, anxious	He wants to return to work but not for the moment
9	♂ 38	Married, no children Works as a technician in an events bureau, producing big shows, theatres, films As the work is located in another city 270 km away he commutes weekly	General tiredness from work and commuting, low energy According to his wife, he is irritable and passive, even aggressive towards his wife	No other complaints	The wife makes the doctor's appointment as he himself has left work three weeks ago and made no contact with his employer

After watching each of the video vignettes, GPs were asked to provide diagnoses and recommend sick leave or no sick leave. GP decisions on sick leave were the main outcomes of the study. Sickness benefits were dichotomized into the dependent variables “sick leave yes” or “sick leave no”. “Sick leave no” was no sickness benefits, and “Sick leave yes” included any of the relevant sickness benefits available at the time of data collection. All countries offered 100% and partial sick leave. In addition, Norway had pending sick leave, medical and vocational rehabilitation, and disability pension, and Sweden had preventive and permanent sick leave.

Participating GPs were asked to respond to statements related to the patients’ health and ability to work on a five-point Likert scale. These were: “From your medical point of view, how long do you think the sick leave period should last?”, “The work situation is the main reason for the patient's complaints”, “His/her private life is the main reason for the patient's complaints”, “Medical and health related factors are the main reasons for granting sick leave”, “The patient is not motivated to work”, “If the patient is not sick listed, the complaints will worsen or the healing process will be slower”, “How would you judge the patient's ability to work?”

### Data analysis

Descriptive statistics were presented as proportions. Between-country differences were tested with ANOVA. A Kruskal–Wallis Test was used to test variance in the length of the sick leave recommended. A one-way ANOVA was used to test variance in use of partial sick leave between countries. To study the effects of the different questionnaire variables on sick leave, a mixed-effects logistic regression model was used. Each registered patient was recorded as a separate observation for each GP, thereby adding an indicator for the GP and an indicator for the patient as random factors. Variables that were statistically significant in country-specific models were entered into a joint model to test for differences in odds ratios (OR) for granting sick leave between the countries. PASW Statistics 18 and the lme4 library in the statistical package “R” [18] was used for the analyses. The results for the mixed model were presented as OR. P-values ≤ 0.05 were considered statistically significant.

## Results

**Figure 1.**
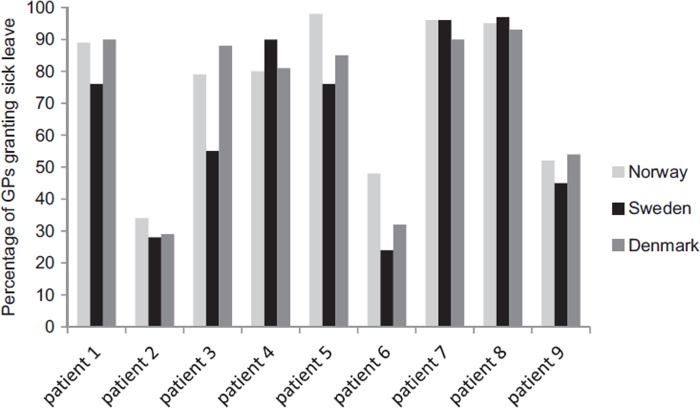
Percentage of GPs in each country granting sick leave to patients 1–9.

The GPs’ sick-leave decisions for the patients presented in the vignettes, were similar between the three countries. Approximately 70% of the GPs recommended sick leave for Patients 1, 3, 4, 5, 7, and 8, but did not do so for Patients 2 and 6. Most variance in the sick-leave decisions was found for Patient 9 where 50% of the GPs suggested sick leave (Figure 1). The median recommended length of sick leave for the nine patients was 2–4 weeks.

Partial sick leave, ranging from 5% to 80%, was recommended for all nine patients by less than one-third of the GPs from Norway and Sweden, and half of the Danish GPs. There were no significant between-country differences.

GPs were significantly more likely to grant sick leave when they agreed with the statement: “If the patient is not sick-listed, the complaints will worsen or the healing process will be slower”, and assessed the patient's ability to work as reduced (Table III). The Norwegian and Swedish GPs were significantly more likely to grant sick leave when they agreed with the statement “Medical and health-related factors are the main reason for sick leave” (Table III). When the Norwegian GPs gave a diagnosis from the ICPC-2 chapters “general and unspecified” or “psychological” they were statistically significantly more likely to grant sick leave compared with other ICPC-2 chapters; however, the confidence intervals were wide (Table III). Being a specialist in general medicine in Norway lowered the OR for granting patients sick leave compared with those with no, or a different medical specialty (Table III).

**Table III.** Adjusted ORs, using mixed-effects logistic regression analysis, of the probability that the GPs in each country will grant the patients sick leave or not.^1.^

	Norway		Sweden		Denmark	
	OR (95% CI)	p-value	OR (95% CI)	p-value	OR (95% CI)	p-value
GP characteristics: Female	1.43 (0.46–4.41)	0.536	1.36 (0.30–6.29)	0.690	0.84 (0.25–2.81)	0.774
Male (ref)	1	–	1	–	1	–
Age Years practicing as a GP Specialist in general medicine	1.01 (0.34–2.98) 1.26 (0.71–2.22) 0.24 (0.06–0.93)	0.985 0.432 0.039	0.87 (0.27–2.74) 1.27 (0.71–2.28) –*	0.809 0.428 –	0.94 (0.31–2.84) 1.24 (0.62–2.46) –*	0.907 0.538 –
No, or other medical specialty (ref) GP's assessment:	1	–	–*	–	–*	–
General and unspecified (A)	7.06 (1.40–35.72)	0.018	–*	–	1.48 (0.30–7.26)	0.629
Psychological (P)	9.94 (2.37–41.78)	0.002	0.33 (0.07–1.61)	0.170	1.52 (0.37–6.19)	0.562
Musculoskeletal (L)	3.20 (0.72–14.33)	0.128	1.63 (0.21–12.77)	0.641	0.41 (0.06–2.80)	0.366
Other organ chapters (ref)	1	–	1	–	1	–
Work situation is the main reason for the patient's complaints	0.90 (0.63–1.30)	0.589	1.23 (0.71–2.12)	0.456	1.46 (0.97–2.19)	0.071
His/her private life is the main reason for the patient's complaints	1.04 (0.65–1.67)	0.870	0.62 (0.30–1.29)	0.200	0.91 (0.58–1.42)	0.674
Medical and health-related factors are the main reason for granting sick leave	0.56 (0.36–0.88)	0.011	0.44 (0.21–0.94)	0.033	0.93 (0.61–1.43)	0.753
The patient is not motivated for work	0.84 (0.56–1.26)	0.407	1.49 (0.85–2.62)	0.161	1.18 (0.80–1.73)	0.408
If this patient is not sick-listed the complaints may worsen or slow down healing	0.29 (0.18–0.46)	< 0.001	0.39 (0.21–0.72)	0.002	0.24 (0.15–0.39)	< 0.001
The patient has reduced work ability	0.30 (0.19–0.46)	< 0.001	0.15 (0.07–0.33)	< 0.000	0.36 (0.21–0.64)	< 0.001
Normal work ability (ref)	1	–	1	–	1	–

Notes: ^1^The model includes characteristics of the GP, the GP's assessment of diagnosis of the patient, and the GP's evaluation of the patient's ability to work, and if work will have a negative effect on the patient's health, and the GP's evaluation of factors that can explain the patient's complaints or need for sick leave. P-values ≤ 0.05 were considered statistically significant. *No estimate due to sparse data or no observations.

### Comparison between countries

GPs from Sweden were less likely to grant sick leave for patients given diagnoses from the “psychological” chapter of the ICPC-2 compared with GPs from Norway (OR = 0.07; 95% CI = 0.01–0.83). There were no other significant findings between the countries.

## Discussion

GPs from Norway, Sweden, and Denmark made similar decisions in their assessments of sick leave, their use of partial sick leave, and their recommendations for the length of sick leave for the vignette patients with severe SHC. The age and gender of the GPs did not influence the decisions to grant sick leave, but being a specialist in general medicine in Norway was associated with granting significantly less sick leave compared with non-specialists and those with a different medical speciality. The decision to grant sick leave appeared to be based on the GP's assessment of the medical condition and health of the patient, the belief that the patient's health would deteriorate if he/she continued working (NO, SE), and the ability of the patient to work. Which diagnosis the GP had chosen was of less importance. However, diagnoses from the ICPC-2 chapters “general and unspecified” or “psychological” were associated with an increase in suggesting sick leave among Norwegian GPs. A “psychological” diagnosis was associated with a significantly lower OR for sick leave among the Swedish GPs, compared with the Norwegian.

The high number of standardized patient stories presented to the GPs provides a reasonably good indication of the type of patients that have severe SHC and their encounters with GPs. The GPs who participated in the study in Norway confirm that these patients are seen frequently in general practice [19]. To our knowledge, this is the first study to use dramatized video vignettes to investigate how GPs make sick-leave decisions. Written vignettes are frequently used in training and education [20] but have been criticized for failing to reflect “real-life” situations [20]. GPs also seem to be more reluctant to grant sick leave based on written vignettes than they are in real life [21]. Observation of a patient's posture and movement, and information about a patient's general appearance are all important in patient assessments [22]. We will argue that the video vignettes offer scenarios that are similar to clinical encounters and allow identical information to be provided to all GPs. It may be argued that one potential weakness of this design is that the GPs were not able to interact with the patients. This may have affected the results. Respondents are inaccurate when predicting or reporting their own behaviour [23]. Despite differences in the organization of primary care in three Scandinavian countries, the assessments conducted by the GPs were very similar.

Recruitment of participants in this study was made by convenience sampling, where the GPs volunteered to participate. The risk of selection bias of GPs with a special interest in patients with severe SHC who participated is thus high. This plausible selection bias applies to all three countries, thus comparison between the countries should not be greatly affected. Clinical experience is likely to influence clinical judgement more than by just being interested in participating in this study and clinical experience varies greatly between the GPs included, and this factor is not considered as selection bias in this study.

Diagnoses have been shown to play a central role in the assessment of ability to work [24,25] and length of sick leave [12,26]. The Swedish GPs in this study were more reluctant to issue sick leave for patients with “psychological” diagnoses. This may be explained by cultural, political, and educational differences [27]. However, the diagnosis-based guidelines for issuing sick leave in Sweden emphasize that patients with “psychological” diagnoses should be encouraged to work, and that long-term sick leave may have negative health consequences [12]. A patient with severe SHC will get different diagnoses depending on which GP they see. One GP may diagnose the same patient with a musculoskeletal diagnosis, while another will give a psychological or gastrointestinal diagnosis on the sickness certificate [14]. Using standardized sick-leave length for a diagnosis may therefore cause changes in which diagnosis the GP gives a patient to accommodate the assessed need for length of sick leave, rather than changes in sick-leave length based on another diagnosis.

Decisions to grant sick leave were based on the assessment of two basic components: if work would be harmful to the patient, or if the patient's ability to work was reduced. This appears to be in line with recommendations in several European countries [11,12,28]. The reported ORs for these two variables are very low and this is because the GPs’ sick-leave decisions were often based on these variables.

Factors such as family or work circumstances, and the motivation to work, were not associated with granting of sick leave. Similar findings have been reported elsewhere [29].

GPs’ age and gender could not explain any differences in granting sick leave. This is in line with other studies [30,31], although there are some conflicting findings [21]. It has been argued that GPs need more training in how to make sick-leave decisions [32], but we have no data on the effect of training GPs. However, specialists in general medicine granted fewer sick leaves than those with no or another medical specialty. This may be due to more clinical experience.

## Conclusion

Overall, sick-leave decisions were similar among the GPs in the three countries. The GPs’ interpretations of the patient's medical condition, health, and ability to work were associated with granting sick leave. Patients given a “psychological” diagnosis were less likely to receive sick leave in Sweden compared with Norway. Having a specialty in general medicine may have an important impact on sick-leave decisions.

## Ethics

The study was approved by the Norwegian Social Science Data Services (project number 20384). The study proposal was evaluated by the Regional Committee for Medical and Health Research Ethics, Western Norway (REC West), and assessed to be outside the mandate of the Committee: the authors were permitted to conduct the study without their approval. The principles in the Helsinki declaration were followed in this study, and the GPs signed a statement confirming their voluntary participation.
